# Development and validation mental training model: Mental Toughness Training Circle (MTTC)

**DOI:** 10.12688/f1000research.129010.1

**Published:** 2023-02-13

**Authors:** Sutoro, Tri Setyo Guntoro, Miftah Fariz Prima Putra

**Affiliations:** 1 Postgraduate School of Sport Education, Universitas Cenderawasih, Jayapura, Indonesia; 2 Department of Sport Sciences, Universitas Cenderawasih, Jayapura, Indonesia

**Keywords:** Mental toughness, mental training, psychological skill, mental toughness training circle, MTTC

## Abstract

**Background**: A systematic and comprehensive mental training program to enhance athletes’ mental toughness is critical. The aim of this study was to develop and validate a set of athletes’ systematic mental training programs.

**Methods**: A mental toughness training program was developed, and the validity and reliability were tested on experts, practitioners, and athletes. Training program was analyzed using content validity index (I-CVI and S-CVI) and modified Kappa ( *k* ^*^). Furthermore, estimation of reliability of mental training model was analyzed by internal consistency approach with Cronbach’s alpha and inter-rater reliability (IRR) approach by using intraclass correlation coefficients (ICC).

**Result**: A mental toughness training circle (MTTC) was successfully developed with four sections: general preparatory, specific preparatory, precompetitive, and competitive with 11 mental skills (positive thinking, mental log, goal-setting, breathing, relaxation, concentration, self-talk, mental imagery, leadership, managing anxiety, and managing emotions). Validation assessment found that the I-CVI and S-CVI values (S-CVI/Ave and S-CVI/UA) were 1.00, each suggesting excellent content validity. The modified Kappa value ( k*) was 1 and categorized as excellent. The results of the reliability test using Cronbach's alpha showed that a value was in the range 0.723 to 0.835 with an overall value of 0.803. The results of the ICC analysis also confirmed that MTTC had a very high reliability coefficient value of 0.803. In addition, there was no significant difference from respondents’ assessment as proved by obtaining value of *F*=0.754 with a p=0.644 (>0.05). This suggested that respondents tend to be consistent in assessing MTTC as a mental training set which was categorized as relevant (scale 3) or very relevant (scale 4).

**Conclusion**: MTTC which has four sections with 11 mental skills is a set of mental training programs that have high quality. Further studies to validate this program in a bigger sample size is required.

## Introduction

The increasing pressure experienced by athletes in the modern era ^1^ has made the topic of mental toughness (MT) popular among scholars. ^2^ Therefore, in the field of sports psychology, studies on the topic of MT have been conducted several times ^3^^,^^4^ and there are still many that can be explored in this area. ^5^ Furthermore, in the history of the development of Mental Skill Training (MST) in sports, the involvement of scientists from the Soviet Union were inseparable. In the 1950s, Avksenty Cezarevich Puni was credited for his work in creating a systematic mental training program. ^6^ The application of systematic mental training by the Soviets to athletes was then followed by other eastern European countries such as Germany and Romania. ^7^ However, because the article was in Russian, the publication received little global attention, thus Ryba and his colleagues ^6^ translated Puni's work into English.

In simple terms, MT describes a person's ability to persevere and focus on reaching specific goals. ^8^ Athletes who have MT will become much better and consistent in determination, focus, confidence, and under pressure management. ^9^^,^^10^ Furthermore, Clough and his colleagues ^11^ developed an instrument to measure MT using four dimensions which were widely known as the 4Cs ( *i.e.*, commitment, challenge, control, and confidence). The work of Clough and his colleagues ^11^ is based on the hardiness theory suggested by Kobasa. ^12^ In contrast to that, Gucciardi *et al.* developed an MT measurement tool using “personality psychology” as a framework. From this work, Gucciardi ^13^ concluded that “MT as a personal capacity to produce consistently high levels of subjective ( *e.g.*, personal goals or strivings) or objective performance ( *e.g.*, sales, race time, GPA) despite everyday challenges and stressors as well as significant adversities.” The other experts, Sheard and his colleagues ^14^ developed MT based on “positive psychology” paradigm as stated by Seligman & Csikszentmihalyi. ^15^

MT can be comprehended as a combination of emotions, attitudes, behaviors, and values that enable individuals to overcome the constraints and pressures they experience while remaining consistent in maintaining motivation and concentration to meet specific goals. ^16^ Although there are many definitions suggested by experts ^10^^,^^13^^,^^17^ these definitions are inconsistent ^18^ and often become conceptual debates. ^13^ Nevertheless, in general MT is a multidimensional concept and is often associated with resolute self-confidence, persistence or capability of bouncing back from failure, capability of dealing with pressure and difficulties effectively, and capability of maintaining concentration despite encountering several distractions. ^19^ Similarly, Gucciardi ^10^ stated that MT generally refers to themes such as belief, focus, motivational drive, control, and regulation of the self ( *e.g.*, thoughts, feelings) during training and competition.

MT is a compatible process and if used in an effective balance can create an optimal pattern to achieve success. ^20^ Athletes with high MT levels are likely to have positive reactions, are better at dealing with pressure ^5^^,^^21^ and show better performance. ^22^ On the other hand, if athletes have low MT level, situations where they face pressure will result in negative reactions such as nervousness, unstable emotions, loss of concentration and behavior beyond the athletes’ internal control. ^11^ Elite athletes are likely to have higher MT level than non-elite athletes. ^23^ Having high MT level can make athletes feel confident, relaxed, calm, and enthusiastic and consistently pursue the target or its purpose.

Based on perspective of sports science, the success or failure of athletes is determined by many complex factors. ^24^ Generally, there are four influential aspects, namely physical, technical, tactical, and psychological. ^19^^,^^25^ That means, in competitive sports it is not only the physical, technical and tactical that have a role but the mental (psychological) aspect of the athletes is also very influential. Indeed, there are many variables that contribute to determining the outcome of a competition, according to Gould *et al.*, ^26^ in world prestigious sport events ( *e.g.* the Olympics), the most influential factor that determines the outcome of a match and athletes’ performance in the field is MT. This happens because at the world elite level, physical and technical factors are relatively the same, considering that athletes have been trained with a variety of training programs and the latest methods. ^27^ Therefore, at that level, it is believed that the athletes’ mental factor contributes greatly in the field. ^26^^,^^28^ US professional tennis player, Alexandra Stevenson, states that “Mental toughness is 90 percent of the game”. ^29^ The same thing was also stated by Australian swimming legend, Elka Graham, that “In training everyone focuses on 90% physical and 10% mental, but in the races it’s 90% mental because there's very little that separates us physically at the elite level”. ^30^ Even though MT has the greatest influence on the outcome of a competition, the facts shows in the field that mental training is often neglected. ^31^^,^^32^ In fact, mental training is very important for athletes, both elite-professional level athletes and beginner level athletes. ^3^^,^^28^^,^^33^

Generally, coaches recognize that the mental aspect influences the success of athletes. ^34^ From the perspective of athletes, especially elite and super elite athletes, they also admit that MT is a vital aspect for them. ^5^^,^^9^ On the other hand, especially in Indonesia, coaches do not prepare specific mental training programs and tend to focus on physical and technical aspects. ^31^^,^^35^ This fact that occurs in Indonesian sports coaching is different from coaching and training overseas which has integrated physical, technical, tactical and mental training in the training menu. ^26^^,^^28^^,^^36^ As matter of fact, the development of modern sports in Indonesia can be seen after Indonesia's independence ^37^ but unfortunately interventions related to the mental aspects of athletes have not been applied much. One of the reasons for this is due to the trainers’ limited knowledge about structured and integrated mental training program for athletes. ^28^ Because of this, the mental training programs are not given to them.

Several models of mental training have been designed by experts. However, we have not found an explanation of how they are carried out systematically and the terms used by experts regarding mental training are also varied. There are those who call it Psychological Skill Training (PST), ^38^^–^^41^ Mental Skill Training (MST), ^16^^,^^42^ Mental Training Skill (MTS) ^43^ and Mental Toughness Training (MTT). ^44^^,^^45^ These terms are often used interchangeably by experts. (see example: ^38^) Therefore, this article does not attempt to differentiate the use of existing terms related to intervention or providing mental training to athletes because we conclude the focus of the goal is the same, namely, to improve the mental quality of athletes or to create a winning mentality.

Mental toughness interventions in athletes have been carried out by experts. ^16^^,^^33^^,^^39^^,^^46^ However, studies on mental training programs applied in the field are still partial. Johnson ^47^ only applied mental training by providing one aspect of the exercise, namely self-talk. Mellalieu ^48^ only used goal setting to improve athletes’ performance. In contrast, other researchers used a set of mental training programs consisting of five psychological skills, ^38^^–^^41^ while Gucciardi ^16^ used two different mental program sets to improve athletes' mental toughness. An expert ^43^ made MTS but unfortunately it has not been tested further and in a wider context. Ragab ^44^ gave MTT to athletes for eight weeks, but the procedures and stages of the mental training program given were not explained in detail. Therefore, we see the need for a mental training program that is arranged systematically and comprehensively to improve athletes' mental toughness. While there are already a set of mental training programs made for athletes, we offer an alternative mental training program that is systematically structured and interrelated. Hence, the purpose of this research is developing and validating systematic mental toughness training programs for athletes.

## Methods

### Ethical clearance

The protocol of the study was approved by the Health Research Ethics Committee, Faculty of Sport Sciences, Universitas Negeri Semarang with the number 266/KEPK/EC/2022. All respondents were requested to provide the written informed consent before participating in this study. Those who received the module and assessment form *via* email and WhatsApp also had to sign the written informed consent prior to participating in the study and return it together with completed assessment form to authors.

### Study design

This research is a type of development research. The development step in this research modifies the procedure proposed by Rodrigues *et al.* ^49^ There are two stages in this study, namely (i) developing a set of mental toughness training program, and (ii) testing the validity and reliability of the model.

### First stage: model development

We started the development step by conducting a literature review. We reviewed articles, e-books, and books related to Mental Toughness (MT). Scientific data search engines ( ScienceDirect, PubMed, and DOAJ) were used to search for relevant literature. Moreover, we also used Google Scholar to add relevant references. We used specific keywords in searching namely: “mental toughness” or “mental training” or “mental skill” or “psychological training” or “psychological skill.” The search was carried out from 15–29 August 2022. Based on the literature review, ^15^^,^^16^^,^^33^^,^^38^^–^^48^ we created a training model to improve athletes’ mental quality or to make athletes have winning mentality (mindset of growth, abundance, and success).

There were two inclusion criteria used for experimental and non-experimental studies (review). For original studies, the criteria for articles that were analyzed further were (1) testing or providing mental training, and (2) using athletes as research samples. For review article, the criteria used were (1) a review of the results of mental training, and (2) discussing mental training programs. Outside of these criteria, the literature will not be analyzed further.

The available references were analyzed descriptively, and we found that: (1) There were researchers who tested the provision of mental training, but this was limited to one mental skill. (2) There were experts who used a set of mental training programs with different number of skills, for example, five mental skills and six mental skill exercises. However, these available set of programs are not comprehensive and still needs to be improved considering some mental skills are still not included in the programs such as positive thinking and breathing. These skills are needed by athletes to build MT. In addition, the training stages for each mental skill are not explained in detail. (3) There were different terms used in giving names to each stage or group of mental training such as categorization into basic mental skills and advanced mental skills, some used the term mental skill types, namely foundation, performance, personal development skills, and team skills. Some refer to them by skill level terms, namely performance skills, preliminary skills, and basic skills. Based on the those results of the available references, we concluded that: (1) the terms in the training program used are relatively unfamiliar to coaches and there was confusion in integrating mental training programs with physical training programs, techniques, and tactics, and (2) there needs to be an alternative mental training program that is more comprehensive, and each mental skill is related to other skills. Therefore, we developed a program called mental toughness training circle (MTTC). In this program, we use terms that are quite familiar to coaches in preparing training programs so far, namely general preparatory, specific preparatory, precompetitive, and competitive. These terms are commonly used by coaches in preparation of training programs.

We then analyzed each skill commonly used by experts and researchers in an effort to shape the MT. In addition, we also looked at the impact of these mental training skills on the psychological aspects of athletes. Taking these aspects into consideration, we found there were 11 mental skills are needed for athletes to have MT, namely positive thinking, mental log, goal-setting, breathing, relaxation, self-talk, concentration, mental imagery, leadership, managing anxiety, and managing emotions. After choosing these eleven skills, we then included them into training component stages that have been made ( *i.e.*, general preparatory, specific preparatory, precompetitive, and competitive). To determine which stage the mental skill belongs to, we decided based on (1) the characteristics of the mental training, (2) the stage or phase of the training, (3) the level of difficulty of the training, and (4) the purpose of the mental skill training. Based on this, we included five mental skills in the general preparatory stage, four in the specific preparatory stage, and two in the precompetitive stage.

### Second stage: model testing

***Participant’s criteria***

After the MTTC training model was successfully developed, we tested the model into four groups of respondents. The criteria of the four groups of respondents involved: (1) sport psychologist who has a Doctoral degree; (2) sport training expert who has Doctoral degree; (3) internationally licensed sport trainers; and (4) outstanding athletes. The internationally licensed sport trainers were trainers who had coached Indonesian national team with Doctoral degree in Sport Sciences or had experiences at national level competitions in Indonesia with a Master degree in Sport Sciences. The outstanding athletes those who had achievements at the national or international level or won a medal at the SEA (Southeast Asian) Games, the Asian Games, or participated in the Olympics.

***Research participants and selection***

The experts were chosen for their educational background, experience, and achievements. There were four criteria for the experts we involved. The experts were selected using non-random sampling technique with purposive sampling. The experts were contacted using phone and we asked them if they were willing to be experts in our research. If they agreed to be experts in this study, the assessment form was provided. Expert judgment is needed so that the model developed has good quality. In end of the study, nine experts participated. As suggested previously ^50^ the recommended number of experts for content validity is between six and ten but no more than ten is recommended. As there were already nine experts who provided ratings, we decided that this was sufficient as it was in line with the recommendation.

The final experts consisted of three sport psychologists who has a Doctoral degree, one sport training experts who has Doctoral degree, three internationally licensed sport trainers; and two outstanding athletes.

***Study instrument and data collection***

To assess the validity of the MTTC model, an assessment form was used to collect data was an assessment form. The assessment form consisted of three parts, namely (1) instructions, (2) mental training model with a brief description of mental training model, and (3) assessment form. The model and the assessment form were handed over to respondents in person or online *via* email and WhatsApp for evaluation and assessment. Five experts participated online and four experts in person. Data collection and analysis were conducted from October 3, 2022 to November 3, 2022.

For each question in the assessment form ^97^, the possible responses were in the form of a Likert scale with a range of 1 (very irrelevant) to 4 (very relevant). The score 1 indicates that the prepared part in the mental training program is not relevant to the concept of the mental training program, while the number 4 indicates that the program is relevant to the concept of the mental training program. The four scales were chosen to avoid having a middle value and this is as suggested by Lynn ^51^ in content analysis. In addition, the assessment sheet also included a “comments” or “suggestions” section to get input from respondents.

The data from the expert assessment ^97^ was entered in Excel by one author. After that, the second author verified the data. The data was coded with numbers one to nine, indicating there were nine experts.

### Data analysis

Research data were analyzed by using Content Validity Index (CVI). CVI is the degree to which an instrument has an appropriate sample of items to construct being measured. ^52^ In this study, CVI analysis was calculated based on each dimension of the mental training program or known as Item-level Content Validity Index (I-CVI) and Scale-level Content Validity Index (S-CVI). ^53^ I-CVI is the Content Validity of individual items or the proportion of content respondents giving the item a relevance rating of 3 or 4. S-CVI is the Content Validity of the overall scale, and is divided into two, namely the proportion of items on a scale that achieves a relevance rating of 3 or 4 by all the respondents (S-CVI/UA) and the average of the I-CVIs for all items on the scale (S- CVI/AVE). S-CVI/UA is a universal agreement calculation method while S-CVI/Ave is an averaging calculation method. ^52^^,^^53^ With a total of nine respondents, the minimum acceptable CVI value is 0.78. ^51^

Although the CVI is widely used by researchers in testing content validity, the index does not consider value increment due to chance agreements. ^54^ Thus, there are experts who suggest that Kappa statistical analysis should also be used ^55^ which Pollit ^51^ later modified the Kappa analysis with the notation k*, namely kappa designating agreement on relevance. Before calculating k*, the probability of a chance occurrence ( *P*c) should be identified, and the following is the formula:

$$p_{c}=\left[\frac{N!}{A!\left(N-A\right)!}\right]∙5^{N}$$

N represents the number of respondents and A represents the number of relevant assessments (scales 3 and 4). After *P*c value is calculated, the analysis progressed by calculating k* using the proportion of agreements on relevance (I-CVI). The formula as follows:

$$k^{*}=\frac{\text{ICVI}-p_{c}}{1-p_{c}}$$

The criteria used to evaluate the value of k* are as follows: Poor= *k*<0.40; Fair= *k* of 0.40 to 0.59; Good= *k* of 0.60–0.74; and Excellent= *k*>0.74. ^56^^,^^57^

The reliability of the mental training model was analyzed by using the internal consistency approach with Cronbach's alpha ^58^^,^^59^ and Inter-Rater Reliability (IRR) approach using the Intraclass Correlation Coefficients (ICC). ^60^^,^^61^ This was chosen because in social studies, Cronbach's alpha and ICC are analysis that are often used by researchers to test reliability. ^59^^,^^61^ The criteria used to interpret the reliability analysis are coefficient values>0.50=low reliability; coefficient values 0.50–0.75=moderate reliability; value>0.75=good reliability. ^58^^,^^61^ Research data analysis was conducted by using IBM SPSS version 26 programs (IBM Corp, Armonk, NY, USA) or the open-access alternative could be STATA (StataCorp LLC, College Station, TX, USA) or R (The R Foundation for Statistical Computing, Vienna, Austria).

## Results

### Development of the MTTC

**Figure 1.**
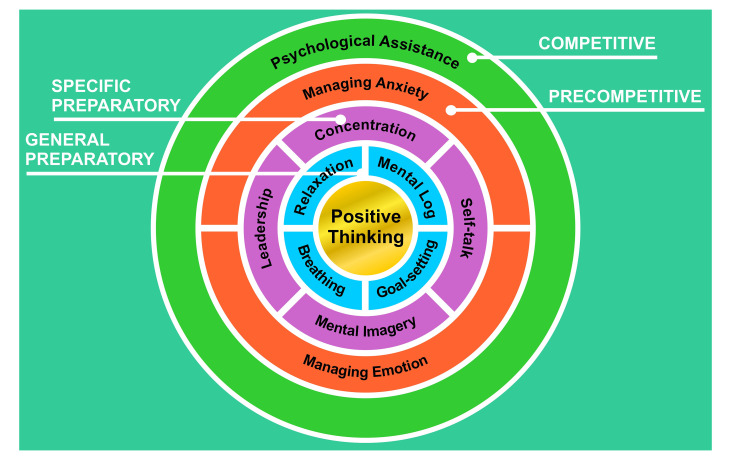
Mental toughness training circle model.

In this study, we have succeeded in developing a systematic mental toughness training program model for athletes. The model created is an integrated training model, which means each psychological skill or mental skill is related to other skills ( Figure 1). There were 11 programs that the researchers developed and each program referred to mental skills that could be used by athletes to improve the quality of their mental toughness. Eleven programs of the mental toughness training circle (MTTC) are presented in Table 1.

**Table 1.** A brief description of the mental training program and the goals for each skill.

No	Mental skills	Training Purpose: *After participating this training, athletes are expected to be able to*:	Days	Time
1	Positive thinking	1. Understand basic concept of mental toughness, namely positive thinking; 2. Understand and identify the benefits of positive thinking for athletes; 3. Provide examples of positive and negative thinking in sport context; 4. Identify the thought process until now, whether it tends to be positive or negative; 5. Practice and get used to positive thinking in daily life.	o Three days of face-to-face practice o Four days of independent practice and habituation	75 minutes X 3 meetings
2	Mental log	1. Understand basic concept of mental toughness, namely mental log; 2. Understand and identify the benefits of making mental log; 3. Provide examples of mental logs made by athletes; 4. Explain and fill in the mental log book; 5. Practice and get used to recording activities and feelings in daily life, especially those related to training and competing.	o Three days of face-to-face practice o Four days of independent practice and habituation	75 minutes X 3 meetings
3	Goal-setting	1. Understand basic concept of mental toughness, namely goal-setting; 2. Understand and identify the benefits of making goal-setting; 3. Provide examples of goal-setting made by athletes; 4. Making goal-setting; 5. Get used to activities written in goal-setting to be achieved in training process and/or matches.	o Three days of face-to-face practice o Four days of independent practice and habituation	75 minutes X 3 meetings
4	Breathing	1. Understand basic concept of mental toughness, namely breathing; 2. Understand and identify the benefits of doing breathing exercise; 3. Practice each type of breathing; 4. Get used to doing breathing exercises.	o Three days of face-to-face practice o Four days of independent practice and habituation	75 minutes X 3 meetings
5	Relaxation	1. Understand basic concept of mental toughness, namely the types of relaxation; 2. Understand and identify the benefits of doing relaxation exercises; 3. Practice each type of relaxation techniques; 4. Get used to doing relaxation exercises.	o Three days of face-to-face practice o Four days of independent practice and habituation	75 minutes X 3 meetings
6	Concentration	1. Understand the basic concept of mental toughness, namely the definition of concentration; 2. Understand and identify the benefits of doing concentration exercise; 3. Practice concentration exercises; 4. Get used to doing concentration exercises.	o Three days of face-to-face practice o Four days of independent practice and habituation	75 minutes X 3 meetings
7	Self-talk	1. Understand basic concept of mental toughness, namely the types of self-talk; 2. Understand and identify the benefits of doing self-talk; 3. Practicing each type of self-talk technique; 4. Get used to doing self-talk exercises.	o Three days of face-to-face practice o Four days of independent practice and habituation	75 minutes X 3 meetings
8	Mental imagery	1. Understand basic concept of mental toughness, namely mental imagery; 2. Understand and identify the benefits of doing mental imagery; 3. Practice mental imagery exercises; 4. Get used to doing mental imagery exercises.	o Three days of face-to-face practice o Four days of independent practice and habituation	75 minutes X 3 meetings
9	Leadership	1. Understand basic concept of mental toughness, namely leadership; 2. Understand and know the benefits of having spirit of leadership; 3. Practice to be a leader; 4. Get used to being a leader for themself and others.	o Three days of face-to-face practice o Four days of independent practice and habituation	75 minutes X 3 meetings
10	Managing anxiety	1. Understand basic concept of mental toughness, namely anxiety; 2. Accept and be aware that anxiety is part of sport; 3. Understand and identify the benefits of being able to manage anxiety; 4. Understand and know how to manage anxiety; 5. Get used to doing anxiety control exercises.	o Three days of face-to-face practice o Four days of independent practice and habituation	75 minutes X 3 meetings
11	Managing emotion	1. Understand basic concept of mental toughness, namely emotion; 2. Accept and be aware that emotions are part of sport; 3. Understand and identify the benefits of being able to manage emotion; 4. Understand and know how to manage emotion; 5. Get used to doing exercises to manage emotion.	o Three days of face-to-face practice o Four days of independent practice and habituation	75 minutes X 3 meetings

MTTC model is in the form of a circle which has four sections: general preparatory consists of positive thinking, daily notes, goal setting, breathing, and relaxation; specific preparatory consists of concentration, self-talk, mental imagery, and leadership; precompetitive consists of managing anxiety and managing emotion; competitive relates to assistance during competition/match. Even though there are four sections, for the mentoring phase, the researchers did not make a specific program, because at that stage the mental training given to athletes would be tested so that the mental coach’s role was as a trainer or just as a person who helped athletes if there were any problems. All of the sections are interrelated and the terms (general preparatory, specific preparatory, precompetitive, and competitive) were deliberately chosen because they conformed to the terminology of training programs that were commonly used by trainers in developing physical training programs, techniques, and tactics (terminology of training programs can be seen in Bompa & Buzzichelli. ^36^ By equating these terms (general preparatory, specific preparatory, precompetitive, and competitive) which was to ascertain that there would be no confusion from the trainers so that the mental training program could be carried out in harmony with the physical, technical, and tactical training programs.

The MTTC model consisted of 11 mental skills that were trained on athletes and each skill was described in three stages, namely: Introduction, Main section, and End section. The introduction section consisted of: pray, apperception, deliver the scope (Scope), and state the objectives (Objective). This section was acronymized for POSE. The Main Section consisted of two types. First type, for the first day of training (beginning of training) each program consisted of: Explaining training menus, Explaining the benefits, Discussions, Instructions, Tasks and Challenges. This section was acronymized for EMBEDDED. Second type, the training on the second and third meetings consisted of: Reflection, Confirmation, and Discussion; acronymized for RECORD. The last part of the training model was the End section. The End section consisted of: Briefly reviewing the material (Highlights), Evaluation, and Pray. This section was acronymized for HAPPY.

### Validity and reliability test of MTTC

After the mental training program was developed, the program was then tested on nine respondents. The results of content validity analysis are presented in Table 2. Based on CVI analysis, both I-CVI and S-CVI (S-CVI/Ave and S-CVI/UN) it appears that the developed program has I-CVI, S-CVI/Ave and S-CVI/UN, each of which has value of 1.00 in the “Appropriate” category and this shows that the program has very high content validity value. The test results using the Kappa modification ( k*) also found very high value of 1 and were included in the “Excellent” category. Based on each stage in the mental skill training program and as a whole, it appears that the program has a very high validity value, based on respondents’ approval score of 9 with a proportion of consensus of 1.

**Table 2.** Analysis result of CVI (I-CVI and S-CVI [S-CVI/Ave; S-CVI/UN]), *P*c, and k*.

No	Mental Training Dimension in MTTC			Relevant (rating 3 or 4)	Not relevant (rating 1 or 2)	I-CVI	Interpretation	*P*c	k*	Interpretation	Comprehensiveness of program dimensions and total program	
											Agree	Proportion of consensus
**1**	**Positive Thinking**										9	1
		**Training purpose**		9	0	1.00	Appropriate	0.001953125	1	Excellent		
	Meeting #1	**POSE**										
	Day: Monday		Pray	9	0	1.00	Appropriate	0.001953125	1	Excellent		
			Apperception	9	0	1.00	Appropriate	0.001953125	1	Excellent		
			Scope	9	0	1.00	Appropriate	0.001953125	1	Excellent		
			Objectives	9	0	1.00	Appropriate	0.001953125	1	Excellent		
		**EMBEDDED**										
			Explanation	9	0	1.00	Appropriate	0.001953125	1	Excellent		
			Benefits	9	0	1.00	Appropriate	0.001953125	1	Excellent		
			Discussion	9	0	1.00	Appropriate	0.001953125	1	Excellent		
			Instructions	9	0	1.00	Appropriate	0.001953125	1	Excellent		
			Task and challenges	9	0	1.00	Appropriate	0.001953125	1	Excellent		
		**HEPPY**										
			Highlights	9	0	1.00	Appropriate	0.001953125	1	Excellent		
			Evaluation	9	0	1.00	Appropriate	0.001953125	1	Excellent		
			Pray	9	0	1.00	Appropriate	0.001953125	1	Excellent		
	Meeting #2	**POSE**										
	Day: Wednesday		Pray	9	0	1.00	Appropriate	0.001953125	1	Excellent		
			Apperception	9	0	1.00	Appropriate	0.001953125	1	Excellent		
			Scope	9	0	1.00	Appropriate	0.001953125	1	Excellent		
			Objectives	9	0	1.00	Appropriate	0.001953125	1	Excellent		
		**RECORD**										
			Reflection	9	0	1.00	Appropriate	0.001953125	1	Excellent		
			Confirmation	9	0	1.00	Appropriate	0.001953125	1	Excellent		
			Discussion	9	0	1.00	Appropriate	0.001953125	1	Excellent		
		**HEPPY**										
			Highlights	9	0	1.00	Appropriate	0.001953125	1	Excellent		
			Evaluation	9	0	1.00	Appropriate	0.001953125	1	Excellent		
			Pray	9	0	1.00	Appropriate	0.001953125	1	Excellent		
	Meeting #3	**POSE**										
	Day: Friday		Pray	9	0	1.00	Appropriate	0.001953125	1	Excellent		
			Apperception	9	0	1.00	Appropriate	0.001953125	1	Excellent		
			Scope	9	0	1.00	Appropriate	0.001953125	1	Excellent		
			Objectives	9	0	1.00	Appropriate	0.001953125	1	Excellent		
		**RECORD**										
			Reflection	9	0	1.00	Appropriate	0.001953125	1	Excellent		
			Confirmation	9	0	1.00	Appropriate	0.001953125	1	Excellent		
			Discussion	9	0	1.00	Appropriate	0.001953125	1	Excellent		
		**HEPPY**										
			Highlights	9	0	1.00	Appropriate	0.001953125	1	Excellent		
			Evaluation	9	0	1.00	Appropriate	0.001953125	1	Excellent		
			Pray	9	0	1.00	Appropriate	0.001953125	1	Excellent		
**2**	**Mental log**										9	1
		**Training purpose**		9	0	1.00	Appropriate	0.001953125	1	Excellent		
	Meeting #1	**POSE**										
	Day: Monday		Pray	9	0	1.00	Appropriate	0.001953125	1	Excellent		
			Apperception	9	0	1.00	Appropriate	0.001953125	1	Excellent		
			Scope	9	0	1.00	Appropriate	0.001953125	1	Excellent		
			Objectives	9	0	1.00	Appropriate	0.001953125	1	Excellent		
		**EMBEDDED**										
			Explanation	9	0	1.00	Appropriate	0.001953125	1	Excellent		
			Benefits	9	0	1.00	Appropriate	0.001953125	1	Excellent		
			Discussion	9	0	1.00	Appropriate	0.001953125	1	Excellent		
			Instructions	9	0	1.00	Appropriate	0.001953125	1	Excellent		
			Task and challenges	9	0	1.00	Appropriate	0.001953125	1	Excellent		
		**HEPPY**										
			Highlights	9	0	1.00	Appropriate	0.001953125	1	Excellent		
			Evaluation	9	0	1.00	Appropriate	0.001953125	1	Excellent		
			Pray	9	0	1.00	Appropriate	0.001953125	1	Excellent		
	Meeting #2	**POSE**										
	Day: Wednesday		Pray	9	0	1.00	Appropriate	0.001953125	1	Excellent		
			Apperception	9	0	1.00	Appropriate	0.001953125	1	Excellent		
			Scope	9	0	1.00	Appropriate	0.001953125	1	Excellent		
			Objectives	9	0	1.00	Appropriate	0.001953125	1	Excellent		
		**RECORD**										
			Reflection	9	0	1.00	Appropriate	0.001953125	1	Excellent		
			Confirmation	9	0	1.00	Appropriate	0.001953125	1	Excellent		
			Discussion	9	0	1.00	Appropriate	0.001953125	1	Excellent		
		**HEPPY**										
			Highlights	9	0	1.00	Appropriate	0.001953125	1	Excellent		
			Evaluation	9	0	1.00	Appropriate	0.001953125	1	Excellent		
			Pray	9	0	1.00	Appropriate	0.001953125	1	Excellent		
	Meeting #3	**POSE**										
	Day: Friday		Pray	9	0	1.00	Appropriate	0.001953125	1	Excellent		
			Apperception	9	0	1.00	Appropriate	0.001953125	1	Excellent		
			Scope	9	0	1.00	Appropriate	0.001953125	1	Excellent		
			Objectives	9	0	1.00	Appropriate	0.001953125	1	Excellent		
		**RECORD**										
			Reflection	9	0	1.00	Appropriate	0.001953125	1	Excellent		
			Confirmation	9	0	1.00	Appropriate	0.001953125	1	Excellent		
			Discussion	9	0	1.00	Appropriate	0.001953125	1	Excellent		
		**HEPPY**										
			Highlights	9	0	1.00	Appropriate	0.001953125	1	Excellent		
			Evaluation	9	0	1.00	Appropriate	0.001953125	1	Excellent		
			Pray	9	0	1.00	Appropriate	0.001953125	1	Excellent		
**3**	**Goal-setting**										9	1
		**Training purpose**		9	0	1.00	Appropriate	0.001953125	1	Excellent		
	Meeting #1	**POSE**										
	Day: Monday		Pray	9	0	1.00	Appropriate	0.001953125	1	Excellent		
			Apperception	9	0	1.00	Appropriate	0.001953125	1	Excellent		
			Scope	9	0	1.00	Appropriate	0.001953125	1	Excellent		
			Objectives	9	0	1.00	Appropriate	0.001953125	1	Excellent		
		**EMBEDDED**										
			Explanation	9	0	1.00	Appropriate	0.001953125	1	Excellent		
			Benefits	9	0	1.00	Appropriate	0.001953125	1	Excellent		
			Discussion	9	0	1.00	Appropriate	0.001953125	1	Excellent		
			Instructions	9	0	1.00	Appropriate	0.001953125	1	Excellent		
			Task and challenges	9	0	1.00	Appropriate	0.001953125	1	Excellent		
		**HEPPY**										
			Highlights	9	0	1.00	Appropriate	0.001953125	1	Excellent		
			Evaluation	9	0	1.00	Appropriate	0.001953125	1	Excellent		
			Pray	9	0	1.00	Appropriate	0.001953125	1	Excellent		
	Meeting #2	**POSE**										
	Day: Wednesday		Pray	9	0	1.00	Appropriate	0.001953125	1	Excellent		
			Apperception	9	0	1.00	Appropriate	0.001953125	1	Excellent		
			Scope	9	0	1.00	Appropriate	0.001953125	1	Excellent		
			Objectives	9	0	1.00	Appropriate	0.001953125	1	Excellent		
		**RECORD**										
			Reflection	9	0	1.00	Appropriate	0.001953125	1	Excellent		
			Confirmation	9	0	1.00	Appropriate	0.001953125	1	Excellent		
			Discussion	9	0	1.00	Appropriate	0.001953125	1	Excellent		
		**HEPPY**										
			Highlights	9	0	1.00	Appropriate	0.001953125	1	Excellent		
			Evaluation	9	0	1.00	Appropriate	0.001953125	1	Excellent		
			Pray	9	0	1.00	Appropriate	0.001953125	1	Excellent		
	Meeting #3	**POSE**										
	Day: Friday		Pray	9	0	1.00	Appropriate	0.001953125	1	Excellent		
			Apperception	9	0	1.00	Appropriate	0.001953125	1	Excellent		
			Scope	9	0	1.00	Appropriate	0.001953125	1	Excellent		
			Objectives	9	0	1.00	Appropriate	0.001953125	1	Excellent		
		**RECORD**										
			Reflection	9	0	1.00	Appropriate	0.001953125	1	Excellent		
			Confirmation	9	0	1.00	Appropriate	0.001953125	1	Excellent		
			Discussion	9	0	1.00	Appropriate	0.001953125	1	Excellent		
		**HEPPY**										
			Highlights	9	0	1.00	Appropriate	0.001953125	1	Excellent		
			Evaluation	9	0	1.00	Appropriate	0.001953125	1	Excellent		
			Pray	9	0	1.00	Appropriate	0.001953125	1	Excellent		
**4**	**Breathing**										9	1
		**Training purpose**		9	0	1.00	Appropriate	0.001953125	1	Excellent		
	Meeting #1	**POSE**										
	Day: Monday		Pray	9	0	1.00	Appropriate	0.001953125	1	Excellent		
			Apperception	9	0	1.00	Appropriate	0.001953125	1	Excellent		
			Scope	9	0	1.00	Appropriate	0.001953125	1	Excellent		
			Objectives	9	0	1.00	Appropriate	0.001953125	1	Excellent		
		**EMBEDDED**										
			Explanation	9	0	1.00	Appropriate	0.001953125	1	Excellent		
			Benefits	9	0	1.00	Appropriate	0.001953125	1	Excellent		
			Discussion	9	0	1.00	Appropriate	0.001953125	1	Excellent		
			Instructions	9	0	1.00	Appropriate	0.001953125	1	Excellent		
			Task and challenges	9	0	1.00	Appropriate	0.001953125	1	Excellent		
		**HEPPY**										
			Highlights	9	0	1.00	Appropriate	0.001953125	1	Excellent		
			Evaluation	9	0	1.00	Appropriate	0.001953125	1	Excellent		
			Pray	9	0	1.00	Appropriate	0.001953125	1	Excellent		
	Meeting #2	**POSE**										
	Day: Wednesday		Pray	9	0	1.00	Appropriate	0.001953125	1	Excellent		
			Apperception	9	0	1.00	Appropriate	0.001953125	1	Excellent		
			Scope	9	0	1.00	Appropriate	0.001953125	1	Excellent		
			Objectives	9	0	1.00	Appropriate	0.001953125	1	Excellent		
		**RECORD**										
			Reflection	9	0	1.00	Appropriate	0.001953125	1	Excellent		
			Confirmation	9	0	1.00	Appropriate	0.001953125	1	Excellent		
			Discussion	9	0	1.00	Appropriate	0.001953125	1	Excellent		
		**HEPPY**										
			Highlights	9	0	1.00	Appropriate	0.001953125	1	Excellent		
			Evaluation	9	0	1.00	Appropriate	0.001953125	1	Excellent		
			Pray	9	0	1.00	Appropriate	0.001953125	1	Excellent		
	Meeting #3	**POSE**										
	Day: Friday		Pray	9	0	1.00	Appropriate	0.001953125	1	Excellent		
			Apperception	9	0	1.00	Appropriate	0.001953125	1	Excellent		
			Scope	9	0	1.00	Appropriate	0.001953125	1	Excellent		
			Objectives	9	0	1.00	Appropriate	0.001953125	1	Excellent		
		**RECORD**										
			Reflection	9	0	1.00	Appropriate	0.001953125	1	Excellent		
			Confirmation	9	0	1.00	Appropriate	0.001953125	1	Excellent		
			Discussion	9	0	1.00	Appropriate	0.001953125	1	Excellent		
		**HEPPY**										
			Highlights	9	0	1.00	Appropriate	0.001953125	1	Excellent		
			Evaluation	9	0	1.00	Appropriate	0.001953125	1	Excellent		
			Pray	9	0	1.00	Appropriate	0.001953125	1	Excellent		
**5**	**Relaxation**										9	1
		**Training purpose**		9	0	1.00	Appropriate	0.001953125	1	Excellent		
	Meeting #1	**POSE**										
	Day: Monday		Pray	9	0	1.00	Appropriate	0.001953125	1	Excellent		
			Apperception	9	0	1.00	Appropriate	0.001953125	1	Excellent		
			Scope	9	0	1.00	Appropriate	0.001953125	1	Excellent		
			Objectives	9	0	1.00	Appropriate	0.001953125	1	Excellent		
		**EMBEDDED**										
			Explanation	9	0	1.00	Appropriate	0.001953125	1	Excellent		
			Benefits	9	0	1.00	Appropriate	0.001953125	1	Excellent		
			Discussion	9	0	1.00	Appropriate	0.001953125	1	Excellent		
			Instructions	9	0	1.00	Appropriate	0.001953125	1	Excellent		
			Task and challenges	9	0	1.00	Appropriate	0.001953125	1	Excellent		
		**HEPPY**										
			Highlights	9	0	1.00	Appropriate	0.001953125	1	Excellent		
			Evaluation	9	0	1.00	Appropriate	0.001953125	1	Excellent		
			Pray	9	0	1.00	Appropriate	0.001953125	1	Excellent		
	Meeting #2	**POSE**										
	Day: Wednesday		Pray	9	0	1.00	Appropriate	0.001953125	1	Excellent		
			Apperception	9	0	1.00	Appropriate	0.001953125	1	Excellent		
			Scope	9	0	1.00	Appropriate	0.001953125	1	Excellent		
			Objectives	9	0	1.00	Appropriate	0.001953125	1	Excellent		
		**RECORD**										
			Reflection	9	0	1.00	Appropriate	0.001953125	1	Excellent		
			Confirmation	9	0	1.00	Appropriate	0.001953125	1	Excellent		
			Discussion	9	0	1.00	Appropriate	0.001953125	1	Excellent		
		**HEPPY**										
			Highlights	9	0	1.00	Appropriate	0.001953125	1	Excellent		
			Evaluation	9	0	1.00	Appropriate	0.001953125	1	Excellent		
			Pray	9	0	1.00	Appropriate	0.001953125	1	Excellent		
	Meeting #3	**POSE**										
	Day: Friday		Pray	9	0	1.00	Appropriate	0.001953125	1	Excellent		
			Apperception	9	0	1.00	Appropriate	0.001953125	1	Excellent		
			Scope	9	0	1.00	Appropriate	0.001953125	1	Excellent		
			Objectives	9	0	1.00	Appropriate	0.001953125	1	Excellent		
		**RECORD**										
			Reflection	9	0	1.00	Appropriate	0.001953125	1	Excellent		
			Confirmation	9	0	1.00	Appropriate	0.001953125	1	Excellent		
			Discussion	9	0	1.00	Appropriate	0.001953125	1	Excellent		
		**HEPPY**										
			Highlights	9	0	1.00	Appropriate	0.001953125	1	Excellent		
			Evaluation	9	0	1.00	Appropriate	0.001953125	1	Excellent		
			Pray	9	0	1.00	Appropriate	0.001953125	1	Excellent		
**6**	**Self-talk**										9	1
		**Training purpose**		9	0	1.00	Appropriate	0.001953125	1	Excellent		
	Meeting #1	**POSE**										
	Day: Monday		Pray	9	0	1.00	Appropriate	0.001953125	1	Excellent		
			Apperception	9	0	1.00	Appropriate	0.001953125	1	Excellent		
			Scope	9	0	1.00	Appropriate	0.001953125	1	Excellent		
			Objectives	9	0	1.00	Appropriate	0.001953125	1	Excellent		
		**EMBEDDED**										
			Explanation	9	0	1.00	Appropriate	0.001953125	1	Excellent		
			Benefits	9	0	1.00	Appropriate	0.001953125	1	Excellent		
			Discussion	9	0	1.00	Appropriate	0.001953125	1	Excellent		
			Instructions	9	0	1.00	Appropriate	0.001953125	1	Excellent		
			Task and challenges	9	0	1.00	Appropriate	0.001953125	1	Excellent		
		**HEPPY**										
			Highlights	9	0	1.00	Appropriate	0.001953125	1	Excellent		
			Evaluation	9	0	1.00	Appropriate	0.001953125	1	Excellent		
			Pray	9	0	1.00	Appropriate	0.001953125	1	Excellent		
	Meeting #2	**POSE**										
	Day: Wednesday		Pray	9	0	1.00	Appropriate	0.001953125	1	Excellent		
			Apperception	9	0	1.00	Appropriate	0.001953125	1	Excellent		
			Scope	9	0	1.00	Appropriate	0.001953125	1	Excellent		
			Objectives	9	0	1.00	Appropriate	0.001953125	1	Excellent		
		**RECORD**										
			Reflection	9	0	1.00	Appropriate	0.001953125	1	Excellent		
			Confirmation	9	0	1.00	Appropriate	0.001953125	1	Excellent		
			Discussion	9	0	1.00	Appropriate	0.001953125	1	Excellent		
		**HEPPY**										
			Highlights	9	0	1.00	Appropriate	0.001953125	1	Excellent		
			Evaluation	9	0	1.00	Appropriate	0.001953125	1	Excellent		
			Pray	9	0	1.00	Appropriate	0.001953125	1	Excellent		
	Meeting #3	**POSE**										
	Day: Friday		Pray	9	0	1.00	Appropriate	0.001953125	1	Excellent		
			Apperception	9	0	1.00	Appropriate	0.001953125	1	Excellent		
			Scope	9	0	1.00	Appropriate	0.001953125	1	Excellent		
			Objectives	9	0	1.00	Appropriate	0.001953125	1	Excellent		
		**RECORD**										
			Reflection	9	0	1.00	Appropriate	0.001953125	1	Excellent		
			Confirmation	9	0	1.00	Appropriate	0.001953125	1	Excellent		
			Discussion	9	0	1.00	Appropriate	0.001953125	1	Excellent		
		**HEPPY**										
			Highlights	9	0	1.00	Appropriate	0.001953125	1	Excellent		
			Evaluation	9	0	1.00	Appropriate	0.001953125	1	Excellent		
			Pray	9	0	1.00	Appropriate	0.001953125	1	Excellent		
**7**	**Concentration**										9	1
		**Training purpose**		9	0	1.00	Appropriate	0.001953125	1	Excellent		
	Meeting #1	**POSE**										
	Day: Monday		Pray	9	0	1.00	Appropriate	0.001953125	1	Excellent		
			Apperception	9	0	1.00	Appropriate	0.001953125	1	Excellent		
			Scope	9	0	1.00	Appropriate	0.001953125	1	Excellent		
			Objectives	9	0	1.00	Appropriate	0.001953125	1	Excellent		
		**EMBEDDED**										
			Explanation	9	0	1.00	Appropriate	0.001953125	1	Excellent		
			Benefits	9	0	1.00	Appropriate	0.001953125	1	Excellent		
			Discussion	9	0	1.00	Appropriate	0.001953125	1	Excellent		
			Instructions	9	0	1.00	Appropriate	0.001953125	1	Excellent		
			Task and challenges	9	0	1.00	Appropriate	0.001953125	1	Excellent		
		**HEPPY**										
			Highlights	9	0	1.00	Appropriate	0.001953125	1	Excellent		
			Evaluation	9	0	1.00	Appropriate	0.001953125	1	Excellent		
			Pray	9	0	1.00	Appropriate	0.001953125	1	Excellent		
	Meeting #2	**POSE**										
	Day: Wednesday		Pray	9	0	1.00	Appropriate	0.001953125	1	Excellent		
			Apperception	9	0	1.00	Appropriate	0.001953125	1	Excellent		
			Scope	9	0	1.00	Appropriate	0.001953125	1	Excellent		
			Objectives	9	0	1.00	Appropriate	0.001953125	1	Excellent		
		**RECORD**										
			Reflection	9	0	1.00	Appropriate	0.001953125	1	Excellent		
			Confirmation	9	0	1.00	Appropriate	0.001953125	1	Excellent		
			Discussion	9	0	1.00	Appropriate	0.001953125	1	Excellent		
		**HEPPY**										
			Highlights	9	0	1.00	Appropriate	0.001953125	1	Excellent		
			Evaluation	9	0	1.00	Appropriate	0.001953125	1	Excellent		
			Pray	9	0	1.00	Appropriate	0.001953125	1	Excellent		
	Meeting #3	**POSE**										
	Day: Friday		Pray	9	0	1.00	Appropriate	0.001953125	1	Excellent		
			Apperception	9	0	1.00	Appropriate	0.001953125	1	Excellent		
			Scope	9	0	1.00	Appropriate	0.001953125	1	Excellent		
			Objectives	9	0	1.00	Appropriate	0.001953125	1	Excellent		
		**RECORD**										
			Reflection	9	0	1.00	Appropriate	0.001953125	1	Excellent		
			Confirmation	9	0	1.00	Appropriate	0.001953125	1	Excellent		
			Discussion	9	0	1.00	Appropriate	0.001953125	1	Excellent		
		**HEPPY**										
			Highlights	9	0	1.00	Appropriate	0.001953125	1	Excellent		
			Evaluation	9	0	1.00	Appropriate	0.001953125	1	Excellent		
			Pray	9	0	1.00	Appropriate	0.001953125	1	Excellent		
**8**	**Mental imagery**										9	1
		**Training purpose**		9	0	1.00	Appropriate	0.001953125	1	Excellent		
	Meeting #1	**POSE**										
	Day: Monday		Pray	9	0	1.00	Appropriate	0.001953125	1	Excellent		
			Apperception	9	0	1.00	Appropriate	0.001953125	1	Excellent		
			Scope	9	0	1.00	Appropriate	0.001953125	1	Excellent		
			Objectives	9	0	1.00	Appropriate	0.001953125	1	Excellent		
		**EMBEDDED**										
			Explanation	9	0	1.00	Appropriate	0.001953125	1	Excellent		
			Benefits	9	0	1.00	Appropriate	0.001953125	1	Excellent		
			Discussion	9	0	1.00	Appropriate	0.001953125	1	Excellent		
			Instructions	9	0	1.00	Appropriate	0.001953125	1	Excellent		
			Task and challenges	9	0	1.00	Appropriate	0.001953125	1	Excellent		
		**HEPPY**										
			Highlights	9	0	1.00	Appropriate	0.001953125	1	Excellent		
			Evaluation	9	0	1.00	Appropriate	0.001953125	1	Excellent		
			Pray	9	0	1.00	Appropriate	0.001953125	1	Excellent		
	Meeting #2	**POSE**										
	Day: Wednesday		Pray	9	0	1.00	Appropriate	0.001953125	1	Excellent		
			Apperception	9	0	1.00	Appropriate	0.001953125	1	Excellent		
			Scope	9	0	1.00	Appropriate	0.001953125	1	Excellent		
			Objectives	9	0	1.00	Appropriate	0.001953125	1	Excellent		
		**RECORD**										
			Reflection	9	0	1.00	Appropriate	0.001953125	1	Excellent		
			Confirmation	9	0	1.00	Appropriate	0.001953125	1	Excellent		
			Discussion	9	0	1.00	Appropriate	0.001953125	1	Excellent		
		**HEPPY**										
			Highlights	9	0	1.00	Appropriate	0.001953125	1	Excellent		
			Evaluation	9	0	1.00	Appropriate	0.001953125	1	Excellent		
			Pray	9	0	1.00	Appropriate	0.001953125	1	Excellent		
	Meeting #3	**POSE**										
	Day: Friday		Pray	9	0	1.00	Appropriate	0.001953125	1	Excellent		
			Apperception	9	0	1.00	Appropriate	0.001953125	1	Excellent		
			Scope	9	0	1.00	Appropriate	0.001953125	1	Excellent		
			Objectives	9	0	1.00	Appropriate	0.001953125	1	Excellent		
		**RECORD**										
			Reflection	9	0	1.00	Appropriate	0.001953125	1	Excellent		
			Confirmation	9	0	1.00	Appropriate	0.001953125	1	Excellent		
			Discussion	9	0	1.00	Appropriate	0.001953125	1	Excellent		
		**HEPPY**										
			Highlights	9	0	1.00	Appropriate	0.001953125	1	Excellent		
			Evaluation	9	0	1.00	Appropriate	0.001953125	1	Excellent		
			Pray	9	0	1.00	Appropriate	0.001953125	1	Excellent		
**9**	**Leadership**										9	1
		**Training purpose**		9	0	1.00	Appropriate	0.001953125	1	Excellent		
	Meeting #1	**POSE**										
	Day: Monday		Pray	9	0	1.00	Appropriate	0.001953125	1	Excellent		
			Apperception	9	0	1.00	Appropriate	0.001953125	1	Excellent		
			Scope	9	0	1.00	Appropriate	0.001953125	1	Excellent		
			Objectives	9	0	1.00	Appropriate	0.001953125	1	Excellent		
		**EMBEDDED**										
			Explanation	9	0	1.00	Appropriate	0.001953125	1	Excellent		
			Benefits	9	0	1.00	Appropriate	0.001953125	1	Excellent		
			Discussion	9	0	1.00	Appropriate	0.001953125	1	Excellent		
			Instructions	9	0	1.00	Appropriate	0.001953125	1	Excellent		
			Task and challenges	9	0	1.00	Appropriate	0.001953125	1	Excellent		
		**HEPPY**										
			Highlights	9	0	1.00	Appropriate	0.001953125	1	Excellent		
			Evaluation	9	0	1.00	Appropriate	0.001953125	1	Excellent		
			Pray	9	0	1.00	Appropriate	0.001953125	1	Excellent		
	Meeting #2	**POSE**										
	Day: Wednesday		Pray	9	0	1.00	Appropriate	0.001953125	1	Excellent		
			Apperception	9	0	1.00	Appropriate	0.001953125	1	Excellent		
			Scope	9	0	1.00	Appropriate	0.001953125	1	Excellent		
			Objectives	9	0	1.00	Appropriate	0.001953125	1	Excellent		
		**RECORD**										
			Reflection	9	0	1.00	Appropriate	0.001953125	1	Excellent		
			Confirmation	9	0	1.00	Appropriate	0.001953125	1	Excellent		
			Discussion	9	0	1.00	Appropriate	0.001953125	1	Excellent		
		**HEPPY**										
			Highlights	9	0	1.00	Appropriate	0.001953125	1	Excellent		
			Evaluation	9	0	1.00	Appropriate	0.001953125	1	Excellent		
			Pray	9	0	1.00	Appropriate	0.001953125	1	Excellent		
	Meeting #3	**POSE**										
	Day: Friday		Pray	9	0	1.00	Appropriate	0.001953125	1	Excellent		
			Apperception	9	0	1.00	Appropriate	0.001953125	1	Excellent		
			Scope	9	0	1.00	Appropriate	0.001953125	1	Excellent		
			Objectives	9	0	1.00	Appropriate	0.001953125	1	Excellent		
		**RECORD**										
			Reflection	9	0	1.00	Appropriate	0.001953125	1	Excellent		
			Confirmation	9	0	1.00	Appropriate	0.001953125	1	Excellent		
			Discussion	9	0	1.00	Appropriate	0.001953125	1	Excellent		
		**HEPPY**										
			Highlights	9	0	1.00	Appropriate	0.001953125	1	Excellent		
			Evaluation	9	0	1.00	Appropriate	0.001953125	1	Excellent		
			Pray	9	0	1.00	Appropriate	0.001953125	1	Excellent		
**10**	**Managing anxiety**										9	1
		**Training purpose**		9	0	1.00	Appropriate	0.001953125	1	Excellent		
	Meeting #1	**POSE**										
	Day: Monday		Pray	9	0	1.00	Appropriate	0.001953125	1	Excellent		
			Apperception	9	0	1.00	Appropriate	0.001953125	1	Excellent		
			Scope	9	0	1.00	Appropriate	0.001953125	1	Excellent		
			Objectives	9	0	1.00	Appropriate	0.001953125	1	Excellent		
		**EMBEDDED**										
			Explanation	9	0	1.00	Appropriate	0.001953125	1	Excellent		
			Benefits	9	0	1.00	Appropriate	0.001953125	1	Excellent		
			Discussion	9	0	1.00	Appropriate	0.001953125	1	Excellent		
			Instructions	9	0	1.00	Appropriate	0.001953125	1	Excellent		
			Task and challenges	9	0	1.00	Appropriate	0.001953125	1	Excellent		
		**HEPPY**										
			Highlights	9	0	1.00	Appropriate	0.001953125	1	Excellent		
			Evaluation	9	0	1.00	Appropriate	0.001953125	1	Excellent		
			Pray	9	0	1.00	Appropriate	0.001953125	1	Excellent		
	Meeting #2	**POSE**										
	Day: Wednesday		Pray	9	0	1.00	Appropriate	0.001953125	1	Excellent		
			Apperception	9	0	1.00	Appropriate	0.001953125	1	Excellent		
			Scope	9	0	1.00	Appropriate	0.001953125	1	Excellent		
			Objectives	9	0	1.00	Appropriate	0.001953125	1	Excellent		
		**RECORD**										
			Reflection	9	0	1.00	Appropriate	0.001953125	1	Excellent		
			Confirmation	9	0	1.00	Appropriate	0.001953125	1	Excellent		
			Discussion	9	0	1.00	Appropriate	0.001953125	1	Excellent		
		**HEPPY**										
			Highlights	9	0	1.00	Appropriate	0.001953125	1	Excellent		
			Evaluation	9	0	1.00	Appropriate	0.001953125	1	Excellent		
			Pray	9	0	1.00	Appropriate	0.001953125	1	Excellent		
	Meeting #3	**POSE**										
	Day: Friday		Pray	9	0	1.00	Appropriate	0.001953125	1	Excellent		
			Apperception	9	0	1.00	Appropriate	0.001953125	1	Excellent		
			Scope	9	0	1.00	Appropriate	0.001953125	1	Excellent		
			Objectives	9	0	1.00	Appropriate	0.001953125	1	Excellent		
		**RECORD**										
			Reflection	9	0	1.00	Appropriate	0.001953125	1	Excellent		
			Confirmation	9	0	1.00	Appropriate	0.001953125	1	Excellent		
			Discussion	9	0	1.00	Appropriate	0.001953125	1	Excellent		
		**HEPPY**										
			Highlights	9	0	1.00	Appropriate	0.001953125	1	Excellent		
			Evaluation	9	0	1.00	Appropriate	0.001953125	1	Excellent		
			Pray	9	0	1.00	Appropriate	0.001953125	1	Excellent		
**11**	**Managing emotion**										9	1
		**Training purpose**		9	0	1.00	Appropriate	0.001953125	1	Excellent		
	Meeting #1	**POSE**										
	Day: Monday		Pray	9	0	1.00	Appropriate	0.001953125	1	Excellent		
			Apperception	9	0	1.00	Appropriate	0.001953125	1	Excellent		
			Scope	9	0	1.00	Appropriate	0.001953125	1	Excellent		
			Objectives	9	0	1.00	Appropriate	0.001953125	1	Excellent		
		**EMBEDDED**										
			Explanation	9	0	1.00	Appropriate	0.001953125	1	Excellent		
			Benefits	9	0	1.00	Appropriate	0.001953125	1	Excellent		
			Discussion	9	0	1.00	Appropriate	0.001953125	1	Excellent		
			Instructions	9	0	1.00	Appropriate	0.001953125	1	Excellent		
			Task and challenges	9	0	1.00	Appropriate	0.001953125	1	Excellent		
		**HEPPY**										
			Highlights	9	0	1.00	Appropriate	0.001953125	1	Excellent		
			Evaluation	9	0	1.00	Appropriate	0.001953125	1	Excellent		
			Pray	9	0	1.00	Appropriate	0.001953125	1	Excellent		
	Meeting #2	**POSE**										
	Day: Wednesday		Pray	9	0	1.00	Appropriate	0.001953125	1	Excellent		
			Apperception	9	0	1.00	Appropriate	0.001953125	1	Excellent		
			Scope	9	0	1.00	Appropriate	0.001953125	1	Excellent		
			Objectives	9	0	1.00	Appropriate	0.001953125	1	Excellent		
		**RECORD**										
			Reflection	9	0	1.00	Appropriate	0.001953125	1	Excellent		
			Confirmation	9	0	1.00	Appropriate	0.001953125	1	Excellent		
			Discussion	9	0	1.00	Appropriate	0.001953125	1	Excellent		
		**HEPPY**										
			Highlights	9	0	1.00	Appropriate	0.001953125	1	Excellent		
			Evaluation	9	0	1.00	Appropriate	0.001953125	1	Excellent		
			Pray	9	0	1.00	Appropriate	0.001953125	1	Excellent		
	Meeting #3	**POSE**										
	Day: Friday		Pray	9	0	1.00	Appropriate	0.001953125	1	Excellent		
			Apperception	9	0	1.00	Appropriate	0.001953125	1	Excellent		
			Scope	9	0	1.00	Appropriate	0.001953125	1	Excellent		
			Objectives	9	0	1.00	Appropriate	0.001953125	1	Excellent		
		**RECORD**										
			Reflection	9	0	1.00	Appropriate	0.001953125	1	Excellent		
			Confirmation	9	0	1.00	Appropriate	0.001953125	1	Excellent		
			Discussion	9	0	1.00	Appropriate	0.001953125	1	Excellent		
		**HEPPY**										
			Highlights	9	0	1.00	Appropriate	0.001953125	1	Excellent		
			Evaluation	9	0	1.00	Appropriate	0.001953125	1	Excellent		
			Pray	9	0	1.00	Appropriate	0.001953125	1	Excellent		
**S-CVI/Ave** = 1.00; **S-CVI/UA** = 1.00												

HAPPY: Highlights, Evaluation, and Pray; I-CVI: Item-Content Validity Index; k*: Modified Kappa; *P*c: probability of a chance occurrence; POSE: Pray, Apperception, Scope, and Objectives; EMBEDDED: Explanation, Benefit, Discussion, Instruction, Tasks and Challenges; RECORD: Reflection, Confirmation, and Discussion; S-CVI: Scale-Content Validity Index; S-CVI/Ave: Scale-Content Validity Item/Average; S-CVI/UA: Scale-Content Validity Item/Universal Agreement.

The reliability test results are presented in Table 3. Analysis using Cronbach's Alpha ( *a*) found the reliability value of each training program ranged from 0.723 to 0.835, whereas the overall reliability value of the training program was 0.803. Using ICC, a value of 0.803 was obtained with a p<0.001. In addition, the results of the respondents’ assessment showed that there was no significant difference, can be proven by the value of *F*=0.754 with p=0.644. These results indicate that the reliability value of the program is very high.

**Table 3.** Reliability test result using Cronbach's Alpha and intraclass correlation coefficients (ICC).

Respondent	Cronbach's Alpha for each respondent	Cronbach's Alpha for all respondents	ICC
1	0.755	0.803	0.803
2	0.775		
3	0.809		
4	0.754		
5	0.826		
6	0.835		
7	0.766		
8	0.723		
9	0.778		

## Discussion

The purpose of this study was to develop and validate athletes’ mental toughness training program. The results of the validity test showed that the developed program had high validity value on both I-CVI and S-CVI (S-CVI/Ave and S-CVI/UA), each of which was 1.00. If the I-CVI value was >0.78 it was considered excellent content validity ^52^^,^^53^ while for S-CVI/Ave a value of 0.90 was included in the category of excellent content validity. ^53^ The same result was found in the Kappa modification value ( k*), which was equal to 1. Given the value >0.74, the validity value was included in the excellent category. ^56^^,^^57^ Thus, it can be stated that the mental training program created has a very good level of content validity.

The content validity value obtained was very good because in the testing process there were no respondents who gave a score of 1 or 2 for each dimension that was assessed in the developed program. Research data showed that respondents tended to give a score 3 or 4 and thus made the CVI score high. This indicates that the respondents have high approval and think that the developed program has good quality.

The results of the reliability test using Cronbach's Alpha showed that the value of *a* was in the range of 0.723 to 0.835 for each dimension, while the overall value of the coefficient *a* was 0.803. The results of ICC analysis also confirmed that the training program that we developed and tested has very high reliability coefficient value, which was 0.803. The reliability coefficient value was categorized as very good ^58^^,^^61^ and was above the minimum value required by Nunnally & Bernstein of 0.70 ^62^ and Fleiss of 0.75. ^63^ Thus, it can be mentioned that the mental training program developed and tested has very good reliability value.

Respondents who examined the MTTC model had similar agreement that no one gave score 1 or 2 but tended to give 3 or 4. This can be proved by obtaining an *F* value=0.754 with p=0.644 which indicated there was no difference based on the respondents’ assessment and the scores given were consistent. Based on this result, it can be stated that respondents tend to assess and perceive that the mental training program that we have developed has very good substance quality.

The results of the study found that there were eleven mental toughness skills which were divided into four sections in the training program, namely general preparatory, specific preparatory, precompetitive, and competitive. The mental toughness training model that we created was different from the model created by Vealey ^64^ which divided the skills into four types ( *i.e.*, foundation skills, performance skills, personal development skills and team skills); Lesyk ^43^ which divided into three levels ( *i.e.*, basic skills, preparatory skills, and performance skills); and Hardy ^65^ divided it into two sections ( *i.e.*, basic and advanced). From this it appears that there is novelty in the phases/stages, the use of terms, and the substance of the mental toughness trainings that researchers have made compared to existing models.

The 11 mental skill exercises that we have developed were broken down into three sections in each program, namely: introduction, main section, and end section. In the introduction section was acronymized for POSE which consisted of Pray, Apperception, Scope, and Objectives. The Main Section consisted of two types. On the first day of training or training at the beginning of each program, it consisted of Explanation, Benefit, Discussion, Instruction, and Tasks and Challenges. This section was acronymized for EMBEDDED. The second part consisted of Reflection, Confirmation, Discussion. This section was acronymized for RECORD. The last part of the training model was the End section, which consisted of Highlights, Evaluation, and Pray. This section was acronym for HAPPY. Thus, if we look at mental training programs such as PST, ^38^^–^^41^ MST, ^15^^,^^41^ MTS ^43^ and MTT, ^43^^,^^44^ the training programs we have developed seemed to be more comprehensive and systematic in training mental skills of athletes.

MTTC is more comprehensive because in the program there were eleven mental skills that used to train athletes. The first training was on positive thinking and our data indicating that each item of within this section had I-CVI value of 1.00 and Kappa modification ( k*) value of 1 suggesting each item was appropriate had excellent criteria. We put positive thinking in the first part because this would be the foundation for the next training process. In fact, our responses or reactions to certain situations is a manifestation of what is in the mind. In our life, we never stop thinking, both consciously and unconsciously. ^66^ The thought of being in a difficult situation can trigger unpleasant or emotional reactions. ^67^ Through the thinking process, people will perceive, then respond accordingly. ^68^ Positive thoughts have massive power in the process of achieving success ^69^ and this is in accordance with the positive psychology approach. ^15^ World basketball legend, Michael Jordan, believed that success is something that comes from the mind. ^70^ Furthermore, he stated that mental toughness is far stronger than physical prowess. ^71^

The second training was on mental logging. Our data found that each item of within this program had I-CVI value of 1.00 and Kappa modification ( k*) value of 1 suggesting each item was appropriate had excellent criteria. Keeping a mental log may seem trivial but we see that this is very important for athletes to do in mental training program. Writing a mental diary is not only useful for recording the athletes’ experience, but more importantly, it can also note sources for correcting what the athletes lack or are weaknesses. ^72^ In the mental log, athletes can write down what is on their minds, images, fears, emotions, and other experiences that are considered important, ^31^ especially those related to training and competition. Therefore, the athletes will have a blueprint for thoughts, feelings and actions to take. ^72^ According to Indonesian sport psychologist, Ali Maksum, the Indonesian badminton legend who had won gold medal in the Olympics and world championship, Susi Susanti, is an example of an athlete who used a mental log. ^72^

The third training was on goal setting and all items were also appropriate had excellent criteria indicating by I-CVI value of 1.00 and Kappa modification ( k*) value of 1. In providing mental training, goal-setting was one of the skills that was often used in sports contexts. ^73^ Thus, expert say that making goal-setting is one of the basics or beginnings in a set of mental training programs. ^74^ A transformation and improvement will occur more easily if athletes formulate or set goals or targets to be achieved. ^75^ Teaching athletes in goal-setting is believed to be important in maintaining and improving the mentality of athletes, especially related to focus and motivation. ^28^ The results of the study prove that athletes receive many benefits from goal-setting. ^76^

The fourth and fifth training was on breathing exercises and relaxation. Our data indicated that all items within these sections had I-CVI value of 1.00 and Kappa modification ( k*) value of 1 suggesting each item was appropriate had excellent criteria. According to experts, breathing exercises are generally associated with relaxation, concentration and meditation, whereas in Indonesia, especially for martial art athletes, breathing exercises are not only used for the above purposes but are also used to raise basic strength or inner strength. ^77^ Thus, breathing exercises will be very helpful in supporting further mental exercises such as relaxation, concentration, mental imagery, managing anxiety, and managing emotions. In addition, to be able to perform all the abilities that have been trained in a competition requires relaxed mental and physical condition. ^31^ Relaxation is a state in which there is no pressure either physically, emotionally or mentally. ^28^^,^^72^ The results of the study show that providing relaxation has positive impact on psychological condition and performance of athletes on the field. ^78^^,^^79^ Hence, the biggest benefit of doing relaxation exercises regularly is that the athletes will have the ability to reduce muscle stiffness due to pressure on their psychological condition. ^28^^,^^75^ If athletes can do relaxation training, this will help the other mental trainings such as concentration training, mental imagery, managing anxiety, and managing emotions.

The sixth and seventh of the training were on concentration and self-talk practices. From our analyses it could be suggested that all items within both sections were appropriate had excellent criteria (I-CVI value and Kappa modification ( k*) value were 1.00 and 1, respectively). Based on literature, the term concentration is often interchanged with attention and focus. ^80^ Concentration is a cognitive functional component ^30^ which plays major role in sports for athletes. ^31^^,^^81^ Thus, concentration is needed for every athlete so that they can perform peak performance. ^28^^,^^82^ Concentration is a state in which a person's awareness is fixed on a certain object at a certain time. ^31^^,^^72^^,^^81^ There are two keywords that can be used to explain concentration, namely “here and now”. ^72^ Hence, a person can be said to have concentration when that person focuses on what he/she faced at that time and in that place, instead of focusing on elsewhere. ^80^ In addition, studies that examine self-talk in sports contexts have found that it has an influence on mood, emotion ^73^ and athletes’ performance. ^83^ Self-talk is divided into two, namely positive self-talk ( *e.g.*, “I am an outstanding athlete”) and negative self-talk ( *e.g.*, “this time I will perform badly again”). ^75^^,^^84^

The eighth training was on mental imagery and our data suggested that each item of within this program was appropriate had excellent criteria. The terms mental imagery and visualization are often used interchangeably, ^27^ despite having differences for an expert. ^30^ Mental imagery can be understood as the ability to use multiple senses to build experiences in the mind by eliminating external stimuli. ^70^ Based on this, there are three aspects that need to be highlighted. First, building experience, namely the athletes’ ability to reconstruct experiences in their mind; second, multi-sensory involvement, namely the athletes’ ability to use the senses of sight, taste, touch, and hearing; and third, eliminating external stimuli, namely the athletes’ ability to minimize distractions that caused while building experiences in their mind. ^70^ Basically, mental imagery can be understood as the athletes’ ability to devise the experiences related to sports performance in their mind. ^85^ The results of the study showed that mental imagery had a positive effect on athletes’ performance. ^86^ Therefore, mental imagery needs to be trained on athletes in a mental training program. ^85^^,^^86^

The ninth training was on leadership and our data suggested that each item of within this program was appropriate had excellent criteria. Basically, leadership can be understood as a person's ability to influence others, both in mind and behavior, to achieve a specific goal. ^28^^,^^64^ The debate about whether leaders were born or can be made has been an issue for a long time. ^87^ However, we do not want to linger on this discourse. In sports, leadership traits are not only needed for coaches, but also for athletes. When athletes have strong leadership within, they will be able to guide themself to focus on a specific goal, namely success or high achievement. Therefore, we made leadership a part of the mental training program for athletes. By leading and developing the spirit of leadership in athletes, we believe these athletes can lead themselves, their teammates, and lead in a wider context, so that opportunities for success become more tangible.

The tenth was on managing anxiety and this section also had appropriate had excellent score from all experts. Anxiety is a normal condition for humans ^30^ and this often appears in the context of sports ^88^^,^^89^ so that the athletes’ anxiety dimension is often studied by associating it to other aspects. ^90^ In general, the factors that cause anxiety for athletes can be grouped into two aspects, namely internal and external factors. ^84^ Then based on criteria or level, anxiety can be divided into three, high, medium and low. Anxiety levels that are too high or too low have a negative impact on athletes’ performances, ^91^ while moderate level of anxiety is believed to have a positive impact on athletes’ performances. ^92^^,^^93^ Therefore, it is important for athletes to be able to manage anxiety properly, so they can show their best performance.

The eleventh was on managing emotion. Our data found that each item of within this program was appropriate had excellent criteria. Basically, emotion is mood condition that is experienced by an individual. ^74^ Emotion can help athletes show their best performance, but on the other hand, it can also make athletes perform far below their abilities. ^94^ Therefore, one of the abilities that athletes must have is the ability to manage emotions. ^32^ There are two types of emotions, namely positive emotion and negative emotion. Positive emotion, for example is feelings of joy and happiness, while example of negative emotion is anger and sadness. Someone who has positive emotion will likely to be flexible, think creatively and find solutions more quickly when faced with problems. ^95^ In competitive sports, there are several reasons that cause athletes to be emotional, for example, the behavior of opponents, referees, coaches, personal problems, upset about their own performance. ^74^ Emotion management training program should be carried out for athletes by informing them that all emotions, both positive and negative, are part of the reality that exists in sports competition. ^43^ Thus, managing emotion needs to be done properly to enhance and improve athletes’ performance. Based on this, it appears that the mental trainings that previously stated can be a solution and to help mastering other mental skills. Thus, we mentioned that these training models were interrelated. Therefore, we conclude that MTTC model training program can be an alternative to provide mental training for athletes.

### Limitations and implications for future research

Even though the systematic and comprehensive mental training program has been developed and has been validated, this research has limitations. First, although the program has been validated by sport psychologists, sports training expert, coaches with national and international achievements, as well as athletes with achievements at national and international levels, the program has not been tested directly on athletes on the field. Therefore, for further research, field testing will be needed to research empirically the effectiveness of the program in developing the mentality of winning athletes. Second, the training program is a set of designs related to mental training for athletes that have been designed systematically. That means, the program does not focus on the individual needs of athletes. In contrast, there are experts who stated that mental training programs need to be developed and modified according to the needs of athletes. ^96^ In other words, interventions or providing mental trainings need to accommodate the needs and conditions of athletes. Third, the program does not explain how to determine the mental toughness of athletes after finishing the training program. In fact, the main purpose of the mental training model is to develop mental qualities. Hence, we recommend tests related to the mental aspects of athletes.

## Conclusions

This research has succeeded in creating a new mental training model called the MTTC model. The results of the validation found that the I-CVI and S-CVI values (S-CVI/Ave and S-CVI/UA) were 1.00 each, which categorized as excellent content validity. For the Kappa modification value ( k*) = 1, which categorized as excellent. The results of the reliability test using Cronbach's Alpha showed that the value of *a* was 0.723 to 0.835 for each program and for the entire training program p=0.803. The results of ICC analysis also confirmed that MTTC had very high reliability coefficient value, which was 0.803. In addition, there was no significant difference from respondents’ assessment which can be proved by obtaining *F*=0.754 with p=0.644. That signifies that, respondents likely to be consistent in assessing MTTC as a mental training set, which then categorized as relevant (scale 3) or very relevant (scale 4). Therefore, this study has contributed to exploring the area of MT and providing practical guidance related to mental training for athletes.

## Data Availability

Figshare: ‘Development and validation mental training model: Mental Toughness Training Circle (MTTC)’. https://doi.org/10.6084/m9.figshare.21628514. ^97^ This project contains the following underlying data:
- Expert Assessment Data.xlsx [Tables containing the raw data of the study] Figshare: ‘Development and validation mental training model: Mental Toughness Training Circle (MTTC)’. https://doi.org/10.6084/m9.figshare.21628514. ^97^ This project contains the following underlying data:
- Module and assessment form-English [Document containing the English version of module and assessment form] - Module and assessment form-Indonesian [Document containing the Indonesian version of module and assessment form] Data are available under the terms of the Creative Commons Attribution 4.0 International license (CC-BY 4.0).
