# Supplementary material for: Development and validation mental training model: Mental Toughness Training Circle (MTTC)
Source: F1000Res. 2023 Feb 13;12:169. [Version 1] doi: 10.12688/f1000research.129010.1 (PMC11389376; doi:10.12688/f1000research.129010.1)
Supplement: Peer review history

# Reviewer report: Reviewer response for version 1

**DOI:** 10.5256/f1000research.141661.r231231

**Published:** 2024-09-09

**Authors:** Rozita Abdul Latif

**Affiliations:** 1 Department of Sport Science and Recreation, Universiti Teknologi MARA, Shah Alam, Selangor, Malaysia; 2 Faculty of Sports Science and Recreation, Universiti Teknologi MARA, Shah Alam, Selangor, Malaysia

The article is relevant, demonstrating the development of a new model in mental training. The generated model is a combination of skills implemented by previous researchers. The article outlines a series of mental training programs developed across four distinct stages with engaging nomenclature to shape a positive mindset. The writing is compelling and easily understandable.  However, it's noteworthy that the use of the term 'HAPPY' differs from the term in Table 2, denoted as 'HEPY'.

Is the work clearly and accurately presented and does it cite the current literature?

Yes

If applicable, is the statistical analysis and its interpretation appropriate?

I cannot comment. A qualified statistician is required.

Are all the source data underlying the results available to ensure full reproducibility?

Yes

Is the study design appropriate and is the work technically sound?

Yes

Are the conclusions drawn adequately supported by the results?

Yes

Are sufficient details of methods and analysis provided to allow replication by others?

Yes

Reviewer Expertise:

socio-psycho, sport sociology, physical education, well-beingl ifestyle

I confirm that I have read this submission and believe that I have an appropriate level of expertise to confirm that it is of an acceptable scientific standard.

# Reviewer report: Reviewer response for version 1

**DOI:** 10.5256/f1000research.141661.r231236

**Published:** 2024-05-08

**Authors:** Vikas Singh

**Affiliations:** 1 Department of Sport Psychology, Lakshmibai National Institute of Physical Education, Gwalior, Madhya Pradesh, India

I've now read the document. Key words and abstract are acceptable. The article will further scholarly discourse and is pertinent to the journal's theme. The report has a clear organisation, and the study's methodology was sound. I think the research problem's topic matter truly noteworthy. Without a doubt, it will advance the pertinent subject of study. The paper's conceptual organisation is noteworthy. Without requiring any major revisions, the work could to be approved.

Is the work clearly and accurately presented and does it cite the current literature?

Yes

If applicable, is the statistical analysis and its interpretation appropriate?

I cannot comment. A qualified statistician is required.

Are all the source data underlying the results available to ensure full reproducibility?

Yes

Is the study design appropriate and is the work technically sound?

Yes

Are the conclusions drawn adequately supported by the results?

Yes

Are sufficient details of methods and analysis provided to allow replication by others?

Yes

Reviewer Expertise:

Educational Leadership, Teaching and Learning,Professional Development, Pedagogy and Education and LearningCurriculum, Pedagogy, Program Development, Sport Performance, Mental Health, Sport Development, Mindfulness-Based Cognitive Therapy, Yoga Research,Yoga as Therapy, and and Meditation

I confirm that I have read this submission and believe that I have an appropriate level of expertise to confirm that it is of an acceptable scientific standard.

# Reviewer report: Reviewer response for version 1

**DOI:** 10.5256/f1000research.141661.r196919

**Published:** 2024-01-02

**Authors:** Ali Maksum

**Affiliations:** 1 Postgraduate School of Sport Science, Universitas Negeri Surabaya, Surabaya, Indonesia

The intention of this research is good but conceptually and methodologically there are weaknesses. First, using the term "circle" in MTTC is inappropriate because the mental training process is a stage or phase, not a circular cycle. Second, it is necessary to clarify the definition of each mental skill so as not to give erroneous and misleading interpretations. Third, the determination of 11 types of mental skills is not based on in-depth analysis. For example, is "leadership" a form of mental skill in athletes? Isn't "breathing" part of relaxation or concentration training? Besides that, determining the type of mental training should be based on an in-depth assessment. Fourth, the placement of certain mental skills at certain stages such as general preparation and pre-competition seems forced. Fifth, the description in Table 2 does not need to be displayed because it is not urgent. Sixth, there is an invalid logic that the main examination of a training model is on its effectiveness in achieving goals, not on content analysis and consistency. Seventh, the mental skills are structured more towards individual sports. What about team or group sports?

Is the work clearly and accurately presented and does it cite the current literature?

Partly

If applicable, is the statistical analysis and its interpretation appropriate?

Not applicable

Are all the source data underlying the results available to ensure full reproducibility?

No source data required

Is the study design appropriate and is the work technically sound?

No

Are the conclusions drawn adequately supported by the results?

Partly

Are sufficient details of methods and analysis provided to allow replication by others?

Partly

Reviewer Expertise:

Sports Psychology

I confirm that I have read this submission and believe that I have an appropriate level of expertise to confirm that it is of an acceptable scientific standard, however I have significant reservations, as outlined above.
